# Characteristics of vestibular corrective saccades in patients with slow visual saccades, vestibular disorders and controls: A descriptive analysis

**DOI:** 10.1371/journal.pone.0197079

**Published:** 2018-05-30

**Authors:** Dario Andres Yacovino, Leigh Alexander Martin, Manuel Perez Akly, Timothy Carl Hain

**Affiliations:** 1 Department of Neurology, Dr. Cesar Milstein Hospital, Buenos Aires, Argentina; 2 Memory and Balance Clinic, Buenos Aires, Argentina; 3 Audiology Department, Interacoustics Academy, Middelfart, Denmark; 4 Northwestern University, Chicago, Illinois, United States of America; Tokai University, JAPAN

## Abstract

**Objective:**

Our aim was to determine whether overt catch up saccades (OS) provoked by vestibular stimuli, as observed in the video head impulse test (vHIT), have comparable metrics as visually triggered horizontal saccades (VS), indicating a common saccadic brainstem generator.

**Methods:**

Three groups of patients were studied: patients with neurological disorders causing slow saccades (group 1, n = 12), patients with peripheral vestibular lesions (group 2, n = 43), and normal controls (group 3, = 24). All patients underwent vHIT and Videooculographic testing. OS velocity, acceleration, amplitude and duration and VS velocity in this group was compared between the groups.

**Results:**

There was significant reduction in the velocity of visually guided saccades in group 1, as expected from the patient selection constraints of this study. Group 1 also exhibited saccades which were longer in duration and of reduced acceleration when compared to subjects without saccadic slowing to visual targets (Group 2 and 3). There were significant positive correlations between OS acceleration and amplitude in both normal saccade groups (2 and 3) which was not observed in the slow saccade group (1).

**Conclusions:**

The metrics of overt saccades measured by the vHIT in patients with slow saccades and normal controls are similar to visually guided saccades. This supports the hypothesis that overt saccades associated with vestibular stimuli and visually triggered saccades share common circuitry that controls metrics.

## Introduction

During self-rotation, vestibular, optokinetic (OKN), smooth pursuit and saccadic eye movements function synergistically for sensory perception. Saccades are fast, brief, and accurate and conjugate eye movements generated by the brainstem. They reposition the fovea onto the visual scene in order to maximize the visual acuity of specific regions of interest [1]. Saccades can be sub classified as being either voluntary or reflexive, and also according to their trigger (e.g. visual, vestibular, sound, etc.).

Visually-guided saccades (VS) are eye movements made in response to the appearance of a visual stimulus. Saccades may also be triggered as a result of prolonged visual or vestibular stimuli that might cause the eye to be driven to the edge of the orbit. These reflexive saccades are called fast phases, and generally move the eyes in the opposite direction with respect to the visual or vestibular input.

When the vestibular stimulus is short and abrupt such as in small and rapid movements of the head, the vestibular ocular reflex (VOR) produces compensatory eye movements in the direction opposite to head movement. This acts to keep visual targets stable on the macula in spite of head movement. However, when the VOR is deficient, the brainstem triggers a rapid ocular movement in the same direction as the VOR, in order to supplement this insufficiency and reduce visual error [2]. This eye movement is called a “catch up” saccade [3].

The video head impulse test (vHIT) quantitatively studies the VOR performance during a rapid head impulse imposed by the examiner [4, 5]. The efficacy of VOR is defined by the either the gain of the slow eye movement to the corresponding head movement or by the presence or absence of systematic corrective (or compensatory) catch up saccades.

Two types of catch up saccades are commonly recognized; overt saccades (OS) and covert saccades (CS). These differ in latency and therefore it is likely they have different mechanisms. Covert saccades have extremely short latency within the range of so-called “express saccades” (<100ms) and occur during the head movement phase. On the other hand, overt saccades have latencies similar to visually guided saccades (150-250ms) [6]. Covert saccades are probably triggered by vestibular signals as vision is obscured during a head impulse. Thus covert saccades may be similar to fast phases. Overt saccades are usually made after the head has come to rest, and thus have a visual trigger, but might also be driven by vestibular signals.

Although they are elicited by different visual stimuli, the dynamic properties of visually guided saccades and quick phases of OKN show similarities that would reflect a shared brainstem substrate [7]. Along the same line, numerous studies have delineated the dynamic properties of different subtypes of saccades in normal subjects [7–10]. In contrast, there is little information that compares the metric properties of saccades triggered by VOR defects with those of visually induced saccades.

Selective slowing of saccades to a visual target is a hallmark of some neurological conditions and forms part of their diagnostic criteria, e.g. Progressive supranuclear palsy (PSP) and spinocerebellar ataxia type 2 (SCA 2) [11, 12]. These conditions are an exceptional model to study the kinematic properties of OS and their relationship with VS.

It is not known if either variant of catch-up saccades, overt or covert associated with VOR deficiency use the same brainstem pathways and have the same vulnerability to lesions as visually guided saccades (voluntary saccades). We hypothesized that both visually triggered saccades and overt saccades are generated by a common mechanism in all patient groups, and therefore VS and OS would exhibit similar metrics. To test this hypothesis, we compared the metrics of OS observed in the vHIT and VS across three well differentiated groups (saccadic generator deficit, peripheral VOR deficit and normal subjects). In addition, to demonstrate the potential clinical applications of our present study, the second goal was to assess if OS metrics are an independent (valuable) measure of saccadic brainstem integrity.

## Materials & methods

### Subjects

A retrospective review of a specialty neuro-otology clinic’s database was performed database between the dates 01/2015 and 06 /2017 was to identify patients with OS during the vHIT test. The following inclusion criteria were satisfied in all patients:

1. A definite neuro-otological diagnosis.
2. A video head-impulse test (vHIT) and video-oculography assessment (VOG), carried out on the same day
3. A vHIT recording with at least 10 impulses to both sides in the horizontal plane and the presence of repetitive overt saccades.
4. A VOG VS evaluation which consisted of a fixed protocol of 20 random visual stimuli to each horizontal side between 0 to 10° (mean 5° +/-2,5°).

Excluded from the study were cases with either of the two features:

1. Incomplete or uninterpretable vHIT/VOG recordings
2. The subject was taking medication that could potentially affect saccadic performance (sedatives, antiepileptic’s, antipsychotics).

Patients with peripheral and/or central conditions were identified and divided into two groups. Group 1 included patients with pathologies associated with a well-established impairment of saccadic generator as diagnostic criteria, resulting in an impaired saccadic velocity [11]. The diagnoses were: Probable (clinically definite without autopsy-confirmed) PSP-Richardson syndrome (PSP-RS) (n = 7), PSP-corticobasal syndrome (PSP-CBS) (n = 1), and SCA 2 (n = 4). Although the most pathognomonic ocular abnormality in PSP is slow vertical saccades, the progression of the disease is also accompanied by cervical antero-posterior rigidness. This resulted in a limitation in reaching sufficient head velocities in vertical head impulses during the vHIT. As a result we decided to use horizontal head impulses and visual guided saccades.

Post-mortem studies were not obtained, however genetic testing was performed in at least in one direct proband in SCA 2 cases. The duration of the illness ranged between 4–10 years. These conditions are well established in the literature as having slowed saccades [13–17].

Group 2 (n = 43) included patients with reduced vestibular function, caused by conditions not associated with an impaired saccade velocity. Well defined peripheral vestibular conditions were included in this group. They were as follows: Unilateral Meniere’s disease treated with gentamicin (n = 3), Vestibular neuritis (n = 28); Bilateral vestibular loss BVL (n = 9), and idiopathic unilateral vestibular loss (n = 3).

Group 3 (n = 24) consisted of a series of asymptomatic healthy subjects that form part of a normative data group in our laboratory. Data for this group was collected between the dates 01/2015–06/2016. Subjects were recruited among clinic personnel, friends and family members of the authors as well as healthy relatives of patients. None had any known neurologic or visual defects other than refractive anomalies. All were clinically evaluated with a neurological and otological bedside examination as well as vestibular testing (VOG, vHIT and vertical subjective measure). It has been documented that OS occur in normal subjects [18]. Our normal subjects generated OS infrequently (12.5% SD 15). When they did occur, they were usually of small amplitude (< 2.3 degrees, mean 1.15). In this study, only those which displayed OS were selected. Overall, 24 subjects (out of a possible 50 and total of 1782 head impulses) with 196 OS were identified and formed part of the analysis along with and their respective VOG saccade data.

Consent was obtained from all subjects verbally, however since this the study is a retrospective analysis of data which was already acquired during diagnostic testing with adequate anonymity the ethics committee considered that consent could be waived.

### Acquisition of data

The VOR was tested in patients using a vHIT system (Interacoustics EyeSeeCam™, Denmark; sample rate 220 Hz). Subjects were placed 1.5 meters from a stationary visual target (1.5 inch diameter red spot on a full white background wall). Head impulses were imposed by the examiner–i.e. the subject’s head was moved at (peak velocity > 150° per second), with small amplitude (10–20 degrees), and in an unpredictable fashion in the horizontal plane. A minimum of 10 impulses were recorded in each direction.

The VOR gains during the vHIT were automatically measured using software that computed the slope of the regression (VORrs) between head and eye velocity. The side-to-side quotient was defined as the asymmetry index (Gain Regression asymmetry). Abnormal VOR gain was defined as below 0.78 for VORrs, and regression asymmetry of > 6% (2 SD) [19, 20].

Since Groups 1 and 2 included both patients with bilateral as well as unilateral peripheral vestibular hypofunction. The average of the right and left VOR gains was used as a total assessment of the vestibular function.

The EyeSeeCam™ software provides sampled video head impulse test data in a MATLAB data file which we used for post hoc analyses. An experienced evaluator (YDA) examined individual head impulse traces using custom software (MATLAB, MathWorks) and rejected head impulses that had pupil-tracking artifact during the head impulse, incorrectly performed head impulses or google slippage. The VOR gains during the vHIT were automatically measured using EyeSeeCam™ software. The regression slope (VORrs) between head and eye velocity was used. Lastly, head thrusts ranging from 180–220 deg/sec of head velocity stimulus were selected.

**Fig 1:**
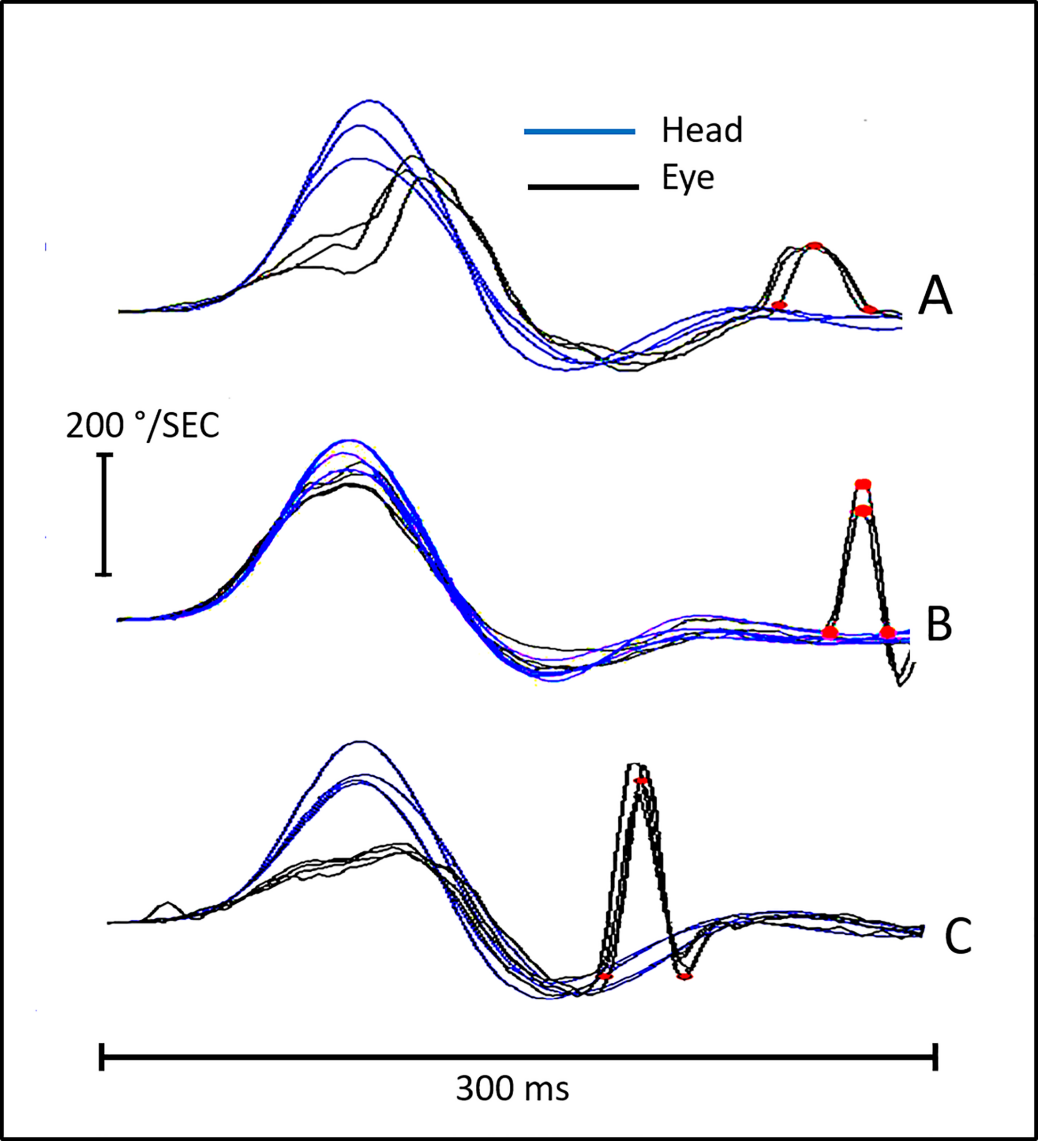
An example of overt saccades generated in patients in each of our 3 groups. A PSP-CBD overlap patient with an abnormally high level of tau protein in cerebrospinal fluid. Mean VS saccades velocity 116°/sec, Mean OS velocity 70°/sec and duration 50 ms. B is a group 3 member—an 84 years old woman normal control, Mean VS saccades velocity 402°/sec, Mean OS velocity 128°/sec and mean OS duration 24,5 ms and In C is a patient with a left sided chronic vestibular neuritis (group 2). Mean VS saccade velocity 420°/sec, Mean OS velocity 241°/sec and mean duration of OS 31 ms. The blue and black curve show head velocity superimposed with eye velocity respectively. Red circles: peak saccade velocity on the top; and duration start and end at the bottom (V0, V1) of the corrective saccades.

OS were defined as quick eye movements occurring after the head velocity returned to 0 degrees/sec. To be classified as an OS these needed to be in the same direction (compensatory) as the VOR and to be rapid eye movements—i.e. having a clearly identifiable acceleration and deceleration distinct from any slower velocity trend line. In addition, they needed to be similarly grouped in terms of their latency and to be repeatable, occurring in greater than 50% of head impulses. Since the aim was to analyze the velocity of the OS, we did not apply any cut-off limits on the velocity or the acceleration. To exclude non-related volitional and erratic saccades we only analyzed the saccades occurring up to 300 ms after head impulse onset, that is once the head velocity is >20°/s as in previous reports [6]. In order to guarantee unique analyzable compensatory eye movements, Covert saccades as well as OS that occurred later than 300ms (up to 500ms) were removed from the traces and only traces with one OS per head movement were considered for further analysis. The base—duration (in ms) and the height–the peak velocity (in °/sec) of up to 5 OS were measured. Since the absolute difference between the base of each saccade was very small (typically < 1ms) the whole number was considered for statistical analysis. The amplitude of each isolated overt saccades was also obtained. Examples of the analysis process for each subject group are shown in Fig 1.

According to the main sequence saccade relationship (see discussion), the peak saccade velocity is a monotonic asymptotic function of the distance the eyes travel. Since saccades exhibit mirror-image symmetry in their acceleration and deceleration for movements up to 10–15° [17]. We decided to measure the average acceleration of OS. This was calculated with the formula: [17] [17]

$$A=\frac{\left(v0-v1\right)}{\left(t/2\right)}\times 1000$$ Eq 1

Where A: saccadic acceleration (°/sec^2^), v0: initial velocity, v1: final velocity, t: duration time of saccade (msec).

The velocity of visually guided saccades (VS) was obtained from the video oculography test (Interacoustics VO425 VOG, Denmark—monocular sample 174 Hz), which was carried out on the same day as the vHIT test. After calibration and an initial training trial, the saccadic test was performed. A low amplitude random (5°+/-2.5) horizontal saccade stimulus was chosen to assess VS velocity. It has been reported that in conditions with slow saccades (e.g. PSP, CBD) and animals (see the discussion), that VS show a complex kinematic combination of hypometric–staircase–acceleration / deceleration and an unstable trajectory [21, 22]. Therefore, we decided to use the mean saccade velocity as the measurement variable of saccade performance for analysis. This measurement is easily recorded and can be applied in a clinical and basic laboratory setting. The VOG device calculated the peak of VS velocity; it is the highest eye velocity in a window limited by a low-high pass filter (90 to 1000°/sec) at the start and end of saccade movement. The individual saccade velocities were plotted and the average velocity was determined.

All tests (VOG and vHIT) were performed by the same neuro-otologist (YDA) with 3 years’ experience in vHIT and about 400 procedures performed annually.

### Statistical analysis

The statistical relationship between vHIT OS velocity, duration, acceleration, amplitude; and VS velocity between groups was measured using the Mann-Whitney U-test. The correlation between OS acceleration and VS velocity as well as between acceleration and amplitude of OS among the three groups was also investigated with Spearman correlation.

Bonferroni correction for multiple comparisons was included. The statistical significance level was adjusted to three groups’ analysis: 0.05 / 24 = 0.002.

Standard Protocol Approval, Registration and Patient Consents. The institutional review board (Dr. Cesar Milstein Hospital, Buenos Aires, Argentina) approved the study protocol.

## Results

Table 1 shows the main results among all three groups, and Table 2 provides the p-values for intergroup comparisons.

**Table 1:** vHIT gains and saccade metricys in all three groups.

	Group 1 (n = 12) Slow visual saccades	Group 2 (n = 43) Vestibular with normal saccades	Group 3 (n = 24) Controls
Mean Age	60.6 (17.3)	61.3 (14.6)	57.7 (22.7)
Ipsilesional side gain †	-	0.12 (0.19)	-
Contralesional side gain †	-	0.86 (0.09)	-
Mean (both side) Gain #	0.8 (0.33)	0.56 (0.20)	0.97 (0.07)
Mean OS duration, ms	33.4 (4.6)	32.4 (5.5)	23.1 (2.5)
Mean OS peak velocity(°/sec)	103.8 (30.5)	206.8 (46.1)	115.0 (19.9)
Mean VS Velocity to the left (°/sec)	210 (72.2)	424 (61.8)	407.7 (32.5)
Mean VS Velocity to the Right (°/sec)	198.8 (49.4)	419.4 (57.9)	411.4 (38.1)
Mean OS Acceleration (°/sec 2)	6199.1 (1359.9)	12855.1 (2398.5)	9969.9 (1002.5)
OS Amplitude (°)	2.05 (0.69)	2.79 (0.96)	1.14 (0.44)

Group 1 includes patients with slow visual saccades, group 2 includes vestibular patients with normal visual saccadic velocity and group 3 includes normal subjects with overt saccades.

† Lesional side VOR gain (vHIT) in a groups of unilateral vestibular lesion (n: 34).

# Both side mean VOR gain are presented for all vestibular, controls and slow visual saccades groups respectively.

**Table 2:** Gain and saccadic metric comparisons between the three study groups.

Variable	Comparison Group 1 vs 2	Comparison Group 1 vs 3	Comparison Group 2 vs 3
Mean Age	0.55	0.39	0.61
Mean Gain	0.006	0.026	0.0001*
Mean OS duration, ms	0.71	0.0001*	0.0001*
Mean OS peak velocity(°/sec)	0.0001*	0.19	0.0001*
Mean VS Velocity to the left (°/sec)	0.0001*	0.0001*	0.16
Mean VS Velocity to the Right (°/sec)	0.0001*	0.0001*	0.53
Mean OS Acceleration (°/sec 2)	0.0001*	0.0001*	0,0001*
OS Amplitude	0.013	0.0001*	0.0001*

Group 1 includes patients with slow visual saccades, group 2 includes vestibular patients with normal visual saccadic velocity and group 3 includes normal subjects with overt saccades. Individual comparison of all main variables between groups is presented on the right of the table. Values in parentheses are SD.

* Significant p<0.002.

There were no statistical differences with respect to the age of subjects among the three groups as well as in the left-right VS velocities among groups. The slow VS patients (group 1) showed the lowest (6199.1 +/- 1359.9) acceleration of OS on the vHIT amongst all groups (p: 0.0001). This group also showed a reduction in VS velocity of around 50% when compared to groups 2 and 3 (p: 0.0001). The only measures where significance was not found between groups 1 and 2 was OS duration (p 0.71) and amplitude (p 0.013) (Table 2).

The VOR gain was lower but not to a significant degree (p 0.026) in patients with slow saccades compared to normal subjects. Unexpectedly however, the peak velocity of OS was not different between groups 1 and 3 (p 0.19). This may reflect a combination of smaller saccades generated by group 3, which would tend to decrease the peak velocity, and poorer VOR gain in group 1 which would tend to increase the peak velocity.

As expected, the largest amplitude OS (mean amplitude = 2.79°) were observed in the vestibular group (2) and the smallest amplitude saccades were seen in the normal group (mean amplitude = 1.14°). No statistical difference was observed in the OS amplitude between group 1 and 2 (p 0.013). In a way similar to velocity of OS results, the reduced VOR gain of the group 1 would tend to increase the OS amplitude in this group.

There was moderate correlation (Rho) between OS acceleration and VS velocity in the group 1: Rho 0.58 (p 0.04). There was no correlation between these variables in the normal group. The correlation between OS acceleration and OS amplitude was not significant in group 1: Rho 0.27 (p 0.4); weak in group 2: Rho 0.32 (p 0.036) and moderate in the group 3: Rho 0.6 (p 0.002). (Table 3).

**Table 3:** Correlations between saccadic parameters in each group.

	Acceleration OS and VS saccades velocity	Acceleration and amplitude OS
Group 1	0.51 (p 0.0001)	0.27 (p 0.4)
Group 2	0.55 (p 0.0001)	0.32 (p 0.036)
Group 3	-0.12 (p 0.58)	0.6 (p 0.002)

Group 1 includes patients with slow visual saccades, group 2 includes vestibular patients with normal visual saccadic velocity and group 3 includes normal subjects

Summarizing the results, OS in the slow VS saccades group (1) were longer in duration, amplitude and had lower acceleration than controls. On the other hand, OS in the peripheral vestibular deficits group (2) showed significantly higher peak velocity, amplitude and acceleration than group 1 and 3, as well as reduced mean VOR gain.

Although the OS amplitudes in the slow saccades group (1) were significantly larger with respect to normal subjects of group 3, the acceleration of OS was significantly inferior (Tables 1 and 2). This emphasizes the large intrinsic differences in group 1’s saccade metrics compared to groups 2 and 3.

## Discussion

We hypothesized that both visually triggered saccades and overt saccades are generated by a common mechanism in all patient groups. To test this hypothesis, we characterized the specific relationships between catch up “overt saccades” in the video head impulse test and visual guided saccades using video-oculography. We observed no statistically significant difference in OS duration between individuals with lesions where VS had abnormally low velocity and peripheral vestibular cases where VS were of normal velocity. However, there were statistically significant differences in mean vHIT metrics of OS including saccade duration, acceleration and mean visual induced saccade velocity in individuals with slow VS (Group 1) compared to those vestibular subjects without slowing and normal controls (Group 2 and 3). To our knowledge, this is the first study to analyze the characteristics of vHIT OS saccade acceleration and velocity.

Saccades are rapid, ballistic movements of the eyes that abruptly change the point of fixation [16]. In addition to their intrinsic function of re-aligning the visual axis to a new novel point of visual interest and sensory perception, they are also considered as the universal non-volitional compensatory eye movement for any reduction in the performance of the ocular motor system (i.e., smooth pursuit or VOR). The movement is considered ballistic because the saccade-generating system cannot respond to subsequent changes in the position of the target during the course of the eye movement. That is, once the saccadic movement is triggered, the metric characteristics (i.e. velocity, acceleration and direction) remain constant, and are often referred to as the “main sequence relationship” [23, 24]. Although saccadic velocity eventually saturates, for saccades that are smaller than 20 degrees there is a linear relationship between amplitude and peak velocity. These movements are generated by the burst neurons of the rostral pons (horizontal saccades) and midbrain (vertical saccades). Saccades are generated by interaction between several brainstem structures, especially the burst cells and superior colliculus cells, and there are several mechanisms that have been postulated as potential sources of slow saccades in animal’s models [22, 25]. Visual error or position bias are considered to be the primary drivers in initiating visually guided saccades. Even though OS during vHIT testing are considered as saccadic movements generated to compensate for the visual or position error between initial and final visual axis displacement in a deficient VOR, a metric correlation to other saccades has not been studied previously and studies to contrast our finding are lacking.

Four of 12 patients in the groups 1 were spinocerebellar ataxia type 2 subjects. Slowed visual guided saccades on horizontal gaze are a hallmark of SCA 2 [26]. Low frequency VOR deficit has been described in about 30% of SCA 2 [26]. However, the occurrence of systematic OS has been observed in 100% of a small series in SCA 2 patients under vHIT analysis, pointing out also some high frequency VOR insufficiency [19]. Recently, Luis et al, studied several groups of Spinocerebellar ataxia patients and controls with vHIT technique. The OS assessment velocity showed significantly lower peak-velocity in SCA 2 [19]. Similarly, in the present study, slow mean peak velocities of OS were observed in the SCA2 patients, supporting a saccadic generator involvement. However, when we expanded the variables to include acceleration, as a more independent value of OS performance, a similar significance was also found.

It is well documented that the video head impulse test is a useful tool to identify VOR deficit due to peripheral vestibular deficits, in particular dysfunction of the semi-circular canals [22]. This study has shown that in patients with slow saccades, namely group 1, the OS induced in the vHIT exhibit the same slowing as VS. When compared to a control group, these patients additionally show a statistically significant reduction in the acceleration of OS. Despite this, there were no significant differences in the duration of the corrective saccades between pathological groups (p: 0.71). This leads to the question—why is it that the saccade duration is not extended to compensate for slower peak velocity in central slow saccades disorders?: Saccades generally have short durations as their role is to move the eye quickly from one position to another to keep the target on the fovea. In healthy subjects the saccadic duration is brief and quite stable according to the main sequence relationship, showing a range of 32 to 48 ms for saccades amplitude of <10° [27]. We hypothesized according to our findings, that under pathological conditions (group 1); the brain is unable to extend the duration of an individual saccade, longer than a maximum limit. The signal that encodes position error when a ballistic saccade is initiated may not be able to be sustained for the longer duration required to move the eye by a smaller population of burst neurons. As was previously postulated by Federighi et al [28], this mechanism might explain the poor fitting of the main sequence and the absence of correlation between OS acceleration and amplitude we observed only in group 1, which would be considered an intrinsic violation of main sequence relationship.

In our series, only 7 of 15 PSP patients generated systematic OS, fulfilling the criteria for inclusion in our study. The reduction of the VOR observed in our study, could represent a peripheral vestibular injury, or vestibular nucleus involvement, or be a finding due to the small numbers of subjects. Although, the low and medium frequency VOR are considered normal or reduced in PSP [29, 30], the high frequency VOR has not been studied. Seeing a reduction of both saccade velocity in the VS test and reduced saccade acceleration in OS of the vHIT implies that VS and OS use the same pathway and may share a common generator.

OS are brief and small amplitude saccades (<5°). In this range, saccade acceleration increases with the amplitude of the saccade [31]. This could explain the higher accelerations in the vestibular group (which have larger OS sizes) with respect to the normal group. Both groups are thought to have a normal saccadic generator. There were statistically positive correlations between OS acceleration and amplitude in both normal saccadic generator groups which was not observed in the slow group 1 (Table 2).

Moving the discussion to the neuro-otologic clinical practice, in addition to VOR gain assessment and systematic OS occurrence, it has been recently suggested that velocity of OS >100°/sec is the cut off limit to consider as one additional abnormal criteria in a vHIT [6, 18]. According to our data the velocity of OS is not only a function of VOR gain but also of brainstem saccadic generator integrity, and thus this criterion would likely fail in subgroups of patients. Qualitative analysis of rapid compensatory movements should be considered when setting norms.

### Limitations

More accurate methods to measure the VS velocity and acceleration such as scleral eye coil systems might provide improved metric data. However, this option was not feasible since our study was performed in the non-invasive clinical setting. Secondly, there were heterogeneous diagnoses in the pathologic groups that could have influenced in the VOR results and consequently in the OS amplitude, however the OS acceleration shown an independent profile and it was well related to the VS velocity. Unfortunately the VS acceleration could not be measured in the VOG device, however in this study we were comparing the kinematic saccades generates by vestibular stimulus in 3 groups (centrally induced slow saccades, peripheral vestibular and normal controls) and VS in the same 3 groups and making the independent correlations. Lastly, should saccade velocity be controlled by other circuitry outside the brainstem, such as the frontal lobes or cerebellum, inferences about a common generator would be unreasonable.

## Conclusion

In this study of groups of patients with slow and normal saccades, we demonstrated that the overt saccades of these patients produced during the vHIT test resemble their visually guided saccades. This supports the conjecture that there is a common brainstem generator. Further work needs to be done to characterize others variables such as latency and precision, to see if they follow the same metric behavior. A possible application for these findings could be testing of children and patients with neurologic disorders who cannot cooperate with saccadic testing of voluntary eye movements. In these patient’s OS analysis can be performed. In the future, a software integrated automatic measure of acceleration of corrective saccades could be a valuable tool.

## Supporting information

S1 File Data sheet of raw data. (XLSX)
